# A review on Gaucher disease: therapeutic potential of β-glucocerebrosidase-targeted mRNA/saRNA approach

**DOI:** 10.7150/ijbs.87741

**Published:** 2024-03-17

**Authors:** Shunping Feng, Nino Rcheulishvili, Xiaoming Jiang, Pan Zhu, Xuehua Pan, Meilan Wei, Peng George Wang, Yang Ji, Dimitri Papukashvili

**Affiliations:** 1 Department of Pharmacology, School of Medicine, Southern University of Science and Technology, Shenzhen 518000, China.; 2 Institute of Microbiology, Chinese Academy of Sciences, Beijing 100101, China.; 3 Cheerland Biomedicine, Shenzhen, China.; 4 Shenzhen Pengbo Biotech Co. Ltd, Shenzhen, China.

**Keywords:** Gaucher disease, mRNA, saRNA, GlcCer, GCase, lysosomal storage disease, ERT, SRT, protein replacement therapy

## Abstract

Gaucher disease (GD), a rare hereditary lysosomal storage disorder, occurs due to a deficiency in the enzyme β-glucocerebrosidase (GCase). This deficiency leads to the buildup of substrate glucosylceramide (GlcCer) in macrophages, eventually resulting in various complications. Among its three types, GD2 is particularly severe with neurological involvements. Current treatments, such as enzyme replacement therapy (ERT), are not effective for GD2 and GD3 due to their inability to cross the blood-brain barrier (BBB). Other treatment approaches, such as gene or chaperone therapies are still in experimental stages. Additionally, GD treatments are costly and can have certain side effects. The successful use of messenger RNA (mRNA)-based vaccines for COVID-19 in 2020 has sparked interest in nucleic acid-based therapies. Remarkably, mRNA technology also offers a novel approach for protein replacement purposes. Additionally, self-amplifying RNA (saRNA) technology shows promise, potentially producing more protein at lower doses. This review aims to explore the potential of a cost-effective mRNA/saRNA-based approach for GD therapy. The use of GCase-mRNA/saRNA as a protein replacement therapy could offer a new and promising direction for improving the quality of life and extending the lifespan of individuals with GD.

## 1. Introduction

Gaucher disease (GD) is a rare, autosomal recessive lysosomal storage disease (LSD) 1 and is one of the most common sphingolipidoses and LSDs 2-6. It was first described in 1882 by French physician Philippe Gaucher, in a 32-year-old female patient with an enlarged spleen 7, and initially was thought to be splenetic cancer 8. GD was better understood in 1965, well after its initial discovery 9. GD is caused by mutations in the *GBA1* gene, encoding the lysosomal enzyme β-glucocerebrosidase (GCase) 1. In contrast, *GBA2* encodes a different, extra-lysosomal enzyme, the so-called second GCase 10,11. It also hydrolyzes GlcCer to glucose and ceramide but operates in a different cellular compartment and is not associated with the lysosomal membrane 12. Unlike GCase, the so-called second GCase is also involved in the metabolism of bile acid 3-O-glucosides 12. The *GBA1* gene, located on chromosome 1 (1q22), is composed of 12 exons (NM_001005742.3) and encodes the enzyme GCase, which consists of 497 amino acids. To date, more than 700 mutations have been detected in this gene in individuals diagnosed with GD 13,14 (https://www.hgmd.cf.ac.uk/ac/gene.php?gene=GBA; http://www.gnomad-sg.org/gene/ENSG00000177628?dataset=gnomad_r3). *GBA1* mutation leads to a markedly decreased activity of the lysosomal enzyme GCase, whose main function is to cleave its substrate glucosylceramide (GlcCer), also called glucocerebroside, into glucose and ceramide, leading to a significant accumulation of GlcCer in macrophages 15. The accumulation occurs in the mononuclear phagocyte system, mainly in spleen histiocytes, bone marrow, lymph nodes, Kupffer cells in the liver, osteoclasts in bone, microglia in the central nervous system (CNS), alveolar macrophages in lungs, etc. 16. Macrophages engorged with unprocessed GlcCer are called Gaucher cells. The infiltration of Gaucher cells in the spleen, liver, and bone marrow is a hallmark of GD, leading to the characteristic manifestations of the disease. These include splenomegaly, hepatomegaly, cytopenia, and bone lesions 7. GD is categorized into three types: GD1, GD2, and GD3. GD1 is the most prevalent (90%) and typically does not involve neurological impairments. In contrast, GD2 and GD3 are associated with neurological damage 15.

The frequency of GD in the general population is approximately between 1 in 40,000 and 1 in 60,000 individuals while it can reach 1 in 800 in the Ashkenazi Jewish population 15,17,18. Once diagnosed, GD typically requires lifetime treatment 19. Enzyme replacement therapy (ERT) and substrate reduction therapy (SRT) stand as the primary treatment modalities for GD1 and partially GD3. Ideally, the patients should receive treatment before the onset of complications 15. Nucleic acid vaccines, especially those based on messenger RNA (mRNA), have recently garnered significant attention within the scientific community. These innovative vaccines represent a potent, cost-effective, time-saving, and safe alternative to conventional immunization strategies for prophylaxis of infectious diseases 20-24. Moreover, preclinical testing of mRNA technology has yielded positive results, and there are presently active clinical trials testing its utility for protein replacement therapy. Yet, there has been a greater emphasis on the development of prevention strategies for infectious diseases. The approval of mRNA vaccines for coronavirus disease 2019 (COVID-19) represents a landmark in the development of this approach, paving the way for new opportunities in managing various health conditions using this technology. Indeed, except for infectious diseases, it has also been widely tested in plentiful directions, including cancer prevention 25. Remarkably, there is an alternative technology to conventional mRNA vaccines which is called self-amplifying RNA (saRNA). Evidently, the saRNA approach represents a cutting-edge advancement in RNA therapy. It is proposed to offer potential improvements over the conventional mRNA technique, particularly due to its lower dosage requirements 26. This may correlate with relatively fewer side effects making it a promising option. Besides, it possesses long-lasting effects which might be a more suitable option for protein replacement therapy 27. Nonetheless, in order to achieve successful saRNA-based drug development and its approval in clinics, some challenges, particularly with its large size and delivery formulations, that exist in this direction need to be overcome 25. In the context of protein replacement therapy, saRNA has the potential to function similarly to the conventional mRNA, yet possibly with enhanced efficiency. A number of advantages observed in the mRNA-based approach 28 also point to a promising future of saRNA-based protein replacement therapy 25.

The current review addresses the strategy for developing a potentially cost-effective mRNA/saRNA-based approach for GD treatment via protein replacement therapy. This will be one step forward to alleviating the disease and improving both life expectancy and quality of life for GD patients. Moreover, the pathophysiology of the disease, its associations with other health disorders, and currently available therapeutics, along with challenges and perspectives will be discussed, providing a holistic view of the current state and potential improvements in GD therapy.

## 2. Pathophysiology

In LSDs, enzyme deficiencies cause substrate accumulation in lysosomes (overload disease) 3. GD is a genetic disorder caused by an enzyme GCase deficiency which is a result of mutations in the *GBA1* gene 13. More than 700 documented gene mutations 13 cause the deficiency of GCase out of which, the most popular mutations are located in N370S, L444P 29, V394L, D409H, K198T, E326K, and R496H 30,31. Additionally, two mutations, E326K and T369M, are strongly related to Parkinson's disease (PD). Interestingly, they are not known to cause GD in homozygous carriers. However, these mutations may still influence the activity of GCase and subsequently alter the clinical presentation of GD 32. GCase which is activated by saposin C 33,34, is responsible for the lysosomal degradation of GlcCer. GCase maturation occurs in the Golgi apparatus from where it is delivered to lysosomes with the assistance of lysosomal integral membrane protein-2 (LIMP-2) molecule 35. After delivering to the lysosome, the molecular bond is broken down via the acidic pH 36. GCase deficiency leads to GlcCer accumulation. The build-up of GlcCer causes the formation of fibrillar aggregates that accumulate in macrophages, giving the cytoplasm the appearance of “crumpled tissue paper” 35. These Gaucher cells infiltrate various organs such as bone marrow, spleen, and liver, and cause the symptoms of the disease 15,17,18. GlcCer is also a substrate of an alternative metabolic pathway where it can be metabolized by acid ceramidase into glucosylsphingosine (GlcSph) which is less hydrophobic and can diffuse into fluids 37. This pathway becomes prominent in the case of GCase deficiency. GlcSph is metabolized by a cytoplasmic enzyme second GCase which is encoded by the *GBA2* gene and functions at neutral pH 15. The so-called second GCase converts GlcSph into sphingosine, which is then phosphorylated, and sphingosine-1-phosphate (S1P) is produced 15. GlcSph, serving as the potential source of S1P, can affect the differentiation, migration, and survival of various cell types, including lymphocytes and macrophages, which may ultimately influence the immune system's function 38. High levels of sphingosine can be toxic to bone 15 while the accumulation of GlcSph can cause neuronal dysfunction and death 15,39. Normally, GlcSph can be detected in the brains of patients with GD-related neurological lesions regardless of the presence of Gaucher cells, while it is absent in the brains of healthy individuals 15 which makes GlcSph a sensitive biomarker 10,40. GlcSph measurements in brain samples of GD patients demonstrate that GlcSph level is remarkably increased in neuronopathic GD, showing the highest levels in GD2 41.

Saposin C, the activator of GCase, is derived by the cleavage of prosaposin- precursor protein into four homologous proteins (saposins A-D) 42. These mature saposins A-D support the activity of lysosomal hydrolases in the process of sphingolipid degradation 42. Saposin C was discovered in 1971, isolated from the spleen of a 12-year-old female patient with GD3. Later research showed its ability to increase GCase activity *in vitro* 42. Apparently, except for the normal functioning of GCase, saposin C also plays a crucial role as the mutation of its gene induces lipid accumulation in lysosomes 43. Those patients carrying saposin C mutation develop biochemical phenotypes mimicking GD 44.

Interestingly, mutations in the *GBA1* gene were also correlated with an enhanced incidence of PD in GD patients and asymptomatic carriers 45. Indeed, the intricate pathophysiology of GD can result in several related conditions, including skeletal abnormalities and PD 7. The skeletal involvement in GD follows three basic processes: focal disease (irreversible lesions such as osteonecrosis and osteosclerosis), local disease (reversible abnormalities adjacent to heavily involved marrow), and generalized osteopenia​ 46. In the general population, the likelihood of developing PD after age 60 is about 2-4%. However, this risk is slightly increased in individuals with GD or those carrying the *GBA* mutation 45,47. These conditions may be a consequence of oxidative stress and inflammatory reactions caused by the interconnection of factors including endoplasmic reticulum (ER) stress, substrate accumulation, defective autophagy, mitochondrial dysfunction, etc. 48.

Infiltration of the spleen by Gaucher cells that exhibit dysregulated expression of surface markers, abnormal release of inflammatory cytokines, and sequestration of iron, leads to splenomegaly. The weight of the spleen in GD patients sometimes reaches several kilograms 49. Indeed, a number of case reports have shown that patients with splenomegaly are often diagnosed with GD and splenomegaly is usually present in most GD patients 50,51. Splenic enlargement which is also reported in patients with chronic kidney disease 52, presents a diagnostic challenge. This similarity of symptoms raises the potential for GD to be misdiagnosed 53. In an *in vitro* model of GD, the deficiency of GCase significantly hampers the process of human bone marrow hematopoiesis 54. In GD patients, an increased occurrence of erythrophagocytosis is observed compared to that in healthy erythrocytes. This phenomenon is associated with an accumulation of sphingolipids within erythrocytes and a corresponding decrease in their deformability 55. Additionally, the impairment of GCase function hampers the degradation of α-Synuclein (α-Syn) in lysosomes, leading to the accumulation of oligomers in the brain- substantia nigra and inducing neurotoxicity 56. On the other hand, the accumulation of GlcCer due to the functional loss of GCase plays a crucial role in the pathogenesis of PD. It modulates the amyloid formation of α-Syn via stabilizing soluble oligomers. Subsequently, these oligomers aggregate and contribute to the formation of Lewy bodies in nerve cells, the hallmark of PD. This process reveals the molecular link between GD and PD 56. Additionally, Moraitou et al. have demonstrated that α-Syn oligomerization takes place in the red blood cell membranes not only in individuals with GD but also in carriers of GD, even in the absence of PD 57. However, in GD patients, α-Syn oligomerization was correlated with lipid abnormalities, while no lipid abnormalities were detected in carriers of GD 57. Thrombocytopenia, characterized by a deficiency of platelets that are crucial for blood clotting following injury, also occurs, leading to coagulation problems in GD patients 53. Gaucher cells also infiltrate the liver in GD patients, frequently leading to hepatomegaly 58. Common manifestations of GD-related liver involvement include hepatomegaly, non-hepatocellular carcinoma (non-HCC) focal liver lesions, as well as fibrosis 59. In more severe cases, GD patients may develop cirrhosis, portal hypertension, and HCC 59.

The abundance of bioactive glycosphingolipids impacts hematopoiesis and disrupts the equilibrium between osteoblasts and osteoclasts in terms of their numbers and activity 60. An imbalance between osteoblasts, the cells responsible for the formation of new bone tissue, and osteoclasts, which break down and resorb bone tissue, contributes to bone thinning, fragility, and the development of osteolytic lesions 60. In general, bone formation and normal functionality are mediated either by hormone receptors which are present on osteoblasts and osteoclasts, or via an indirect way which implies the presence of cytokines including interleukin (IL)-1, IL-6, and tumor necrosis factor-α (TNF-α) 61. Hence, the cytokines are also responsible for the regulation of osteoclasts and osteoblasts 61. Apparently, the changes in cytokine release in GD are associated with different conditions. For example, IL-10 activity, the release of which is elevated in GD, may lead to the inhibition of osteoblast activity while elevated levels of IL-6 in GD are thought to enhance osteoclast activation and formation 61. Additionally, evidence suggests that systematically increased levels of S1P due to the activity of sphingosine kinase (SphK) 1 or SphK2, in the bloodstream may increase the risk of fractures associated with osteoporosis 62. Moreover, elevated S1P may be considered a biomarker in bone disease 62.

The lung involvement in GD is associated with the infiltration of Gaucher cells into the lung leading to an interstitial disease process that has the potential to progress to pulmonary fibrosis, pulmonary arterial hypertension, or reduction of lung volume due to hepatosplenomegaly 63,64.

**Figure 1:**
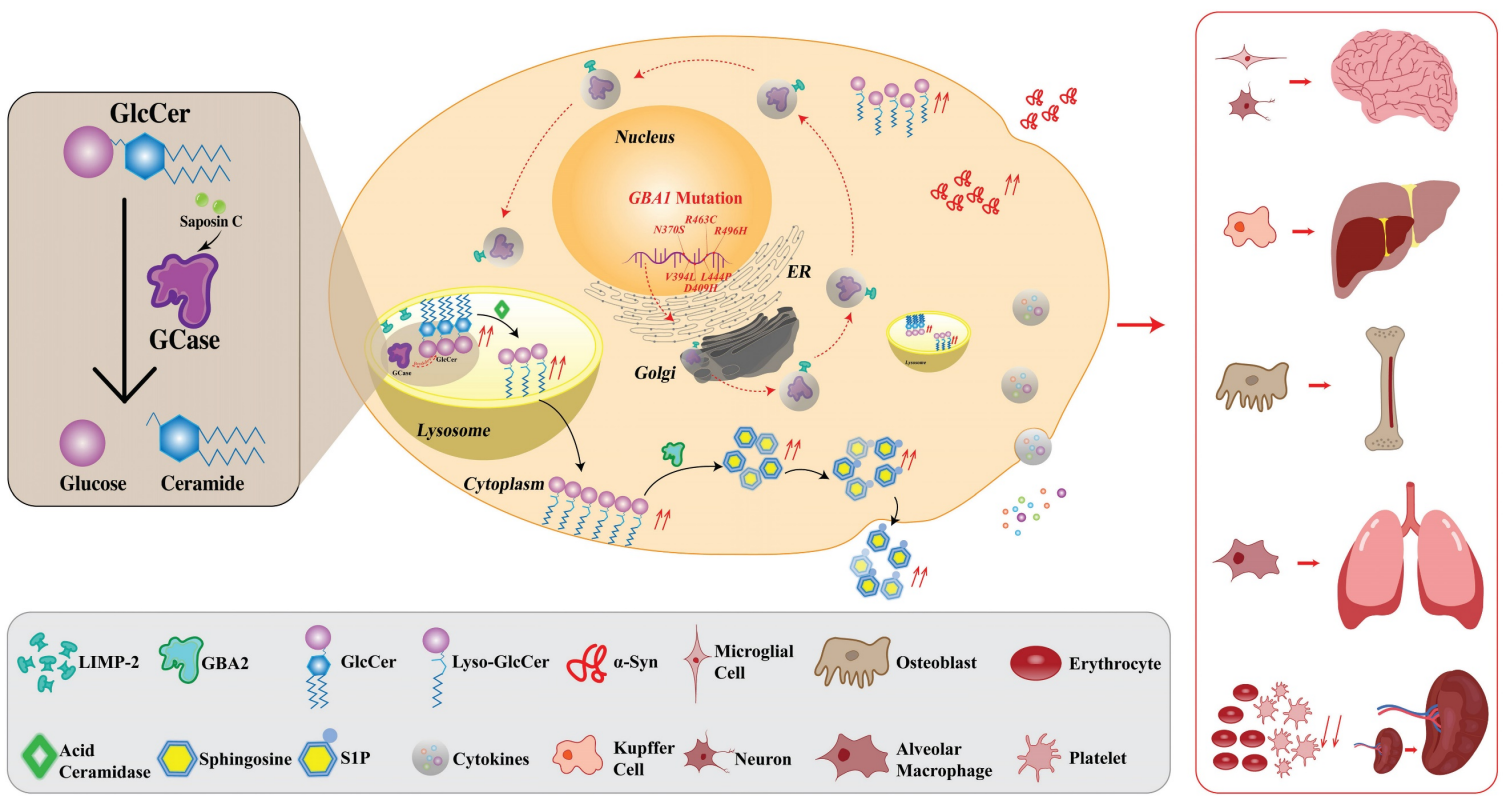
**Pathophysiology of GD in macrophage.** Normally, *GBA1* is transcribed into mRNA which is subsequently exported from the nucleus to the ER where GCase is synthesized. GCase binds to LIMP-2 which is essential for the regulation of GCase transport from ER through the Golgi apparatus (where the GCase protein undergoes maturation) to the lysosomes. The binding occurs at a neutral pH and dissociation takes place within the lysosomal environment, which is characterized by an acidic pH. In the lysosome, with the support of its essential co-factor saposin C, GCase catalyzes the hydrolysis of GlcCer into glucose and ceramide under an acidic environment. In the GD condition, due to the *GBA1* mutation, the mutant GCase is produced which is a target for ERAD and proteasomal breakdown. Mutant GCase is unable to reach the lysosome, leading to an accumulation of GlcCer within the lysosomal compartment. The upregulated levels of intracellular GlcCer promote the formation of toxic soluble α-Syn assemblies, exacerbating a pathogenic cycle by impairing the maturation of lysosomes and inhibiting the activity of functional GCase. This impairment results in further GlcCer accumulation and augmented α-Syn formation due to its hampered degradation, leading to the release of inflammatory cytokines. The enzyme known as GBA2 (so-called second GCase) transforms GlcSph (also known as lyso-GlcCer) into sphingosine, which subsequently undergoes phosphorylation, resulting in the production of S1P. Enhanced inflammatory response, along with the associated pathophysiology, contributes to bone fragility, lowering the erythrocyte and platelet levels, reducing lung function, and may also lead to neurodegeneration. Black arrows represent reactions that proceed normally. The red broken arrows indicate the reactions that are typically expected to occur but do not occur. The red solid arrows denote the reactions that take place, but they are harmful. GD, Gaucher disease; GCase, glucocerebrosidase; LIMP-2, lysosomal integral membrane protein 2; ER, endoplasmic reticulum; ERAD, endoplasmic reticulum-associated degradation; GlcCer, glucosylceramide; α-Syn, α-Synuclein; GBA2, β-glucocerebrosidase 2; S1P, sphingosine-1-phosphate; lyso-GlcCer, lyso-glucosylceramide.

Apparently, except for the GCase deficiency, the anomalies of other implicated members of this molecular cascade such as saposin C or LIMP-2, may also induce GD phenotypes. However, the deficiency of GCase remains the most prevalent and fundamental leading to the manifestation of GD. A schematic representation of the pathophysiology of GD is provided in Fig. 1.

The GD has been classified into three forms according to the absence (GD1) or presence and severity (GD2 or GD3) of neurological involvement 53. These three types can be somewhat but not absolutely distinguished by their severity, symptoms, and age of onset. There are also perinatal-lethal and cardiovascular forms of GD 53. The symptoms vary from a few or asymptomatic forms of the disease to chronic and severe complications 53. Visceral involvement and bone disease are commonly observed to some extent across all forms of GD with the potential for symptomatic overlap among the different types 65. In specific circumstances, the distinctions between the three forms of GD may become unclear. Although GD1 is clinically non-neuronopathic, in some cases, patients develop neurological symptoms 66. Besides, some patients exhibit an intermediate phenotype that falls between GD2 and GD3 with a survival range of 3 to 8 years 67. The classification and characterization of GD are given in Table 1.

### 2.1. GD1

Each type of GD has distinct clinical features, severity levels, as well as prognoses, with GD1 being the most common and milder form as it commonly does not affect the CNS. GD1 usually does not involve the nervous system due to the presence of functional GCase in the brain 15,68. It accounts for 90% of all types of GD cases and is generally characterized by a slower progression 69. The mutation in N370S of *GBA1* is responsible for approximately 70% of GD1 cases in Ashkenazi Jews 70. GD2 and GD3, especially GD2, are less prevalent as they are characterized by higher rates of mortality at an early age 68. Even though there is a higher occurrence of GD1 among Ashkenazi Jews, the majority of patients with this type of disease are non-Jewish individuals, considering the relatively small size of the Ashkenazi Jewish population on a global scale 65. The clinical phenotype of GD1 is widely acknowledged for its exceptional variability 10. GD1 primarily affects the organs such as the liver and spleen 15. Patients with GD1 generally have a normal life expectancy compared with the other two types of GD 71. The clinical presentation varies widely among individuals and mostly includes organomegaly, bone marrow expansion, osteopenia resulting in bone degeneration and deformities, thrombocytopenia, as well as anemia due to the low levels of red blood cells 72. While CNS involvement is atypical in GD1, a minority of GD1 patients may develop peripheral neuropathy 73. Besides, GD1 patients are at risk for developing Lewy body-associated parkinsonism 48.

### 2.2. GD2

GD2, an acute neuronopathic form, is the most severe and rapidly progressive type of the disease, typically manifesting in infancy, in the first year of life, with fatal outcomes 71. It affects the brain and nervous system, causing developmental delays, seizures, and muscle rigidity 74. Most children with GD2 do not survive beyond early childhood 71,75. In GD2, Gaucher cells are not only found in the perivascular spaces of the brain 76, which are pial-lined, fluid-filled structures 76, but are also present as free parenchymal Gaucher cells within the cerebral cortex 77. Notably, there is a particular prevalence of these cells in the occipital lobes 77. Farfel-Becker et al. used a mouse model of GD2 and observed that when the continuous neuronal accumulation of GlcCer reaches a certain level, a rapid cascade of neuroinflammation and neurodegeneration is induced in particular regions of the brain and may lead to neuronal cell death 78,79. The symptoms of GD2 include progressive cognitive decline, muscle stiffness, loss of motor skills, difficulty in coordination, respiratory difficulties, and swallowing disorders along with the systemic manifestation of GD 80,81. There are several phenotypes associated with GD2: hydrops fetalis- a condition when an excessive buildup of fluid in various parts of the body occurs; hepatosplenomegaly; skin abnormalities including congenital ichthyosis and abnormal, cellophane-like skin; dysmorphology of face features 82; neurological involvements such as joint contractures, decreased body movements, etc.; thrombocytopenia and anemia 80. Lal et al., have analyzed data from 23 GD2 patients (3 living, 20 deceased) 83. The span of ages at the time of death varied from 3 to 55 months, with an average age of 19.2 months. Despite the aggressive therapeutic interventions, the condition of the fourteen patients receiving ERT, the two treated with SRT, and the three who underwent bone marrow transplantations, worsened along with the disease progression. Apparently, while the current therapeutics can aid in prolonged life, they are unable to repair neurological complications 83. Thus, GD2 remains a profoundly severe and progressive disorder leading to early mortality, underscoring the urgent need for innovative and effective therapies.

### 2.3. GD3

GD3, also called chronic neuronopathic GD, falls between GD1 and GD2 in terms of severity and usually begins in childhood during the first decade of life. It affects both the visceral organs and the CNS, and the severity of symptoms varies widely 84. GD3 includes patients with neurological manifestations that do not align with the criteria for GD2, representing a highly heterogeneous and phenotypically diverse subgroup 35. Symptoms of GD3 may include enlargement of the spleen and liver, bone abnormalities, eye movement problems, progressive neurological deterioration, and cardiovascular calcification 65. The clinical manifestation of GD3 can vary widely; some affected individuals may only survive their teenage years or early 20s, while others live for much longer. As the condition progresses and symptoms intensify, individuals may require assistance with daily tasks. GD3 has a higher incidence compared to GD2 due to the significantly longer lifespan of individuals affected by the disease 85. While GD is found among different ethnicities, specific clusters of the disease have been extensively studied in Northern Europe, Egypt, and Eastern Asia 65. Because of the common symptoms across different types of GD, a misdiagnosis may take place. For instance, a child with GD3 may not exhibit any neurological symptoms until reaching adolescence, leading to an initial misdiagnosis as GD1 65.

## 3. Associations of GD with other diseases

Apart from its primary manifestations, GD is associated with the increased risk and incidence of other health issues. These conditions include PD 86,87, bone complications 46,60, blood disorders 88, and multiple myeloma 89. Additionally, individuals with GD may experience increased susceptibility to infections and cancer.

### 3.1. Dementia with Lewy bodies

Dementia with Lewy bodies (DLB) is a condition characterized by a gradual deterioration of cognitive functions. The clinical data suggest that there is a strong association between the *GBA1* mutation and the DLB 90. These data indicate that the patients carrying *GBA1* mutations exhibit a more severe phenotype across disorders with Lewy bodies. These individuals tend to experience symptoms at an earlier age, display intensified motor and cognitive impairments, and have a higher incidence of visual hallucinations as well as rapid eye movement (REM) sleep disorders 90. Mutations of the *GBA1* gene are indeed associated with the increased risk of synucleinopathy and lead to DLB via the accumulation of intracellular α-Syn oligomers in the neurons 91,92.

### 3.2. Parkinson's disease

PD is a slowly advancing neurological disorder that primarily impacts the dopamine-producing neurons responsible for controlling body movements. Although GD1 is considered a non-neuronopathic form of the disease, having it increases the risk of developing PD 93. The association of GD1 with PD has posed a challenge to the established classification paradigm 94. About 5-10% of PD patients have *GBA1* mutations 86,87 (https://pdgenetics.shinyapps.io/gba1browser/). The accumulation of α-Syn deposits- Lewy bodies in the brain, in the substantia nigra region that is responsible to produce dopamine, may contribute to the development of PD 95. In addition, mutations in the *GBA1* gene, which are associated with GD, have been identified as a risk factor for PD in the general population 96. Studies have also suggested that individuals with GD who develop PD may have a more aggressive form of the disease compared with individuals without GD 95. Alcalay et al. studied the GCase activity in blood samples obtained from 392 PD patients and 175 healthy controls 97. There was a significant correlation between the *GBA1* status and GCase activity. Particularly, GCase activity was decreased in *GBA1* p.E326K PD carriers compared to the control carriers 97. Remarkably, patients with PD without *GBA1* mutations also have lowered levels of GCase 98. Along with the E326K, T369M has been also associated with an increased risk for PD, suggesting that while it may not directly cause GD, it could contribute to the risk of developing PD 99.

α-Syn is a protein that is encoded in humans by the gene *SNCA* 100. The mutation of the *SNCA* gene is involved in GD as well as the increased risk of developing PD 100. When the GlcCer accumulation takes place in GD, it elevates levels of the α-Syn protein that contributes to the neurodegeneration and development of symptoms resembling PD 101. Hence, mutations in the *SNCA* gene are potentially associated with both GD and PD. Nevertheless, further research is essential to fully understand the connection between these conditions 102-104. Current evidence suggests a relationship between GCase deficiency and the accumulation of α-Syn, a protein critically involved in the pathogenesis of PD 105.

### 3.3. Multiple myeloma

Multiple myeloma is a type of blood cancer when abnormal growth of monoclonal plasma cells in the bone marrow takes place 106. The clinical manifestations include cytopenia and bone lesions that are common for GD as well 89. Individuals with GD have an increased risk of developing multiple myeloma, especially when they have severe bone involvement 107. The exact mechanism of this association is not well understood, but it is thought to be related to immune dysfunction and chronic inflammation associated with GD 107. Indeed, Taddei et al., have evaluated the risk of cancer in 403 patients and found that within the entire cohort, there was an unparallel rise in the lifetime risk of multiple myeloma, primarily observed among N370S homozygous patients 108.

### 3.4. Gammopathy

Noteworthily, gammopathies which imply conditions with abnormal antibodies in the blood are also associated with GD 88. Multiple myeloma is associated with the wide occurrence of monoclonal gammopathy of undetermined significance (MGUS) which is a condition characterized by the presence of monoclonal gammopathy with uncertain implications 109. A multicenter study investigating 2123 GD1 patients in terms of the cancer risk and gammopathies demonstrated that multiple myeloma after MGUS diagnosis was 7.9% which is similar to that observed in the general population 88. Noteworthily, GD1 patients had a higher risk of developing solid cancers of the liver (2.9 times), melanoma (2.5 times), breast (1.4 times), and kidney (2.8 times) 88.

### 3.5. Bone manifestations

Osteoporosis is a condition characterized by bone loss and fragility as a result of bone demineralization. Individuals with GD are at increased risk of developing osteoporosis due to the accumulation of GlcCer in the bone marrow 60. The accumulated GlcCer interferes with the normal function of bone cells, leading to bone loss and an increased risk of fractures 46,60. Fortunately, the treatment with ERT has been shown to improve bone density in GD patients, reducing the risk of osteoporosis 46.

## 4. Current therapeutics

The main therapeutics for GD include ERT and SRT. ERT has been the primary treatment for GD for many years and has been shown to improve bone density, liver and spleen size, and hematological parameters. SRT, on the other hand, is a newer therapeutic option that works by inhibiting the synthesis of the lipids that accumulate in GD 110. The main challenges associated with both therapies include the high cost of ERT and the limited effectiveness of SRT in certain patient populations 53. ERT remains the standard of care for GD, however, new treatment options such as gene therapy and pharmacological chaperones may provide additional benefits in the future 111. Throughout the course of the 15-year retrospective study, a total of 60 patients with various forms of GD were observed where the median age of diagnosis was 2 years. The ERT and SRT could increase the survival of GD patients 110. The Food and Drug Administration (FDA)-approved therapies for GD are given in Table 2.

### 4.1. ERT

ERT is a well-established treatment for GD which involves intravenous (I.V.) administration of a recombinant form of the deficient enzyme GCase. In general, ERT seems to be effective for the treatment of several LSDs, including GD, Fabry disease, and Pompe disease 112. In GD, the administered enzyme is taken up by the lysosome in the affected cell and helps to break down the accumulated substrate GlcCer 111. Indeed, ERT has been shown to ameliorate the condition of various clinical manifestations of GD, including organomegaly, hematological abnormalities, and bone disease 113. Besides, it has demonstrated improvements in quality of life and survival in GD patients 113. In 1991, ERT was introduced as a treatment for GD, which realized the vision initially proposed by Roscoe Brady in 1966 114,115. Currently, several ERT products are approved by the FDA and are commercially available for GD treatment (Table 2). These ERTs are imiglucerase (Cerezyme, Sanofi-Genzyme), velaglucerase alfa (VPRIV, Takeda), and taliglucerase alfa (Elelyso, Pfizer) produced via Chinese hamster ovary cells, human fibroblasts, and carrot cells, respectively 111. The results of these three ERT products are promising. Indeed, treatment with imiglucerase demonstrated exceptional and long-lasting efficacy in GD1 patients while minimal toxicity was observed 116. Other two ERTs- velaglucerase and taliglucerase have also demonstrated remarkable efficacy, safety, and long-lasting effects 117,118. Despite the effectiveness of ERT, the treatment does have some limitations. These include the requirement of lifelong I.V. administration and the production of exogenous recombinant GCase antibodies which can decrease the bioavailability of the enzyme and impact clinical outcomes. Besides, the treatment faces challenges due to the short half-life of the enzyme *in vivo*, the high cost of the treatment, and the insufficient efficacy for all manifestations of the disease 119,120. Notably, ERT is less effective in treating severe neurological symptoms due to the inability to cross the blood-brain barrier (BBB) and reach the CNS 121. Hence, for GD2, a rare and severe form of the disease, ERT has been found to be ineffective. Therefore, alternative treatment approaches are necessary for GD, such as substrate reduction therapy or gene therapy.

### 4.2. SRT

The pathogenesis of LSDs at a cellular level is complex and not fully understood yet. While some LSDs can be treated with ERT, there are also small molecule therapies such as SRT and chaperone therapy. In addition, gene therapy and genome editing are in advanced preclinical stages and have already made their way to clinical trials for a few disorders 122.

ERT aids the body in recycling more waste material, specifically GlcCer, whereas SRT helps the body generate less of this waste material. SRT does not rely on enzyme replacement but instead reduces the accumulation of glycolipids by balancing the residual activity of the deficient enzyme with lowered substrate levels 123. Recent studies have shown that SRT- oral miglustat, which also can cross BBB 124, may be more effective than ERTs in reducing the size of the spleen and liver, improving bone density, and reducing levels of the biomarker chitotriosidase 123. SRT venglustat with the potential of crossing BBB can alleviate the symptoms and prevent disease progression 125. On the other hand, miglustat has low effectiveness and can cause several side effects 125. For this reason, it has been approved for use in a limited population of adults with GD1 for whom ERT is not feasible 126. As to another SRT eliglustat, the study demonstrated that after a one-year treatment, it is not inferior to ERT 127. Furthermore, the long-term study showed that individuals treated with eliglustat maintained stability for up to four years 128.

### 4.3. Chaperone therapy

A molecular chaperone is a type of protein that assists in the folding, unfolding, assembling, and disassembling of other proteins in the cell 129. Pharmacological chaperone therapy is an approach that employs small-molecule ligands to selectively stabilize mutant enzymes, elevate their levels within cells, and enhance their activity and lysosomal trafficking 129. The stabilized enzyme is then able to function properly and break down the GlcCer that accumulates in the body of GD patients. It is noteworthy that both miglustat and eliglustat, apart from their inhibitory effect on the synthesis of GlcCer, have also been shown to increase GCase activity by serving as chaperones. Consequently, they help in reducing the levels of GlcCer 130. Pharmacological chaperones have been also developed as an alternative therapy for GD-associated PD 131. N-Octyl-b-valienamine is the first chaperone used for GD. It has been found to enhance the activity of F213I mutant GCase in cultured cells 132. Another chaperone for GD- ambroxol has been evidenced to have a therapeutic effect on bone and hematological symptoms in child with GD1 133. Moreover, its high dose and long-term application demonstrated promising results in GD1 patient with hepatic fibrosis 134. There are three clinical studies focused on chaperone therapy (NCT01463215, NCT03950050, NCT04388969). Although, chaperone therapy exhibits great promise for GD treatment, its mutation-specific nature limits its applicability. Indeed, Ivanova et al., have shown that the ambroxol increased the GCase activity in cells of patients with L444P/L444P; RecΔ55, RecNciI, and L444P/D409H *GBA1* mutations while it could not have positive effects on GCase activity in cells with L444P/L444P, D409H, A456P mutations 130.

### 4.4. Gene therapy

Gene therapy is a sophisticated modality for treating genetic disorders such as GD, involving the strategic introduction of a functional copy of the defective gene to compensate for the genetic aberration 135. This technique encompasses two primary methodologies: *in vivo* and *ex vivo* gene therapy. *In vivo* gene therapy entails the systemic delivery of a therapeutic gene directly into the patient 136, leveraging vectors such as adeno-associated viruses (AAVs) which are notable for their capability to transverse the BBB- a critical consideration for neurological manifestations of GD 137. The choice of vector is paramount, especially in targeting the central nervous system, where the BBB presents a formidable barrier to many therapeutic agents. AAV vectors, in particular, are engineered for their neurotropic characteristics, enabling them to infiltrate the BBB and deliver therapeutic gene to the affected neuronal cells 138. Conversely, *ex vivo* gene therapy involves transfecting patient-derived cells with the corrective gene exogenously, followed by the transplantation back into the patient 136. The vectors of choice for this method are typically retroviral 139 or lentiviral vectors 140, each with unique integration properties and long-term expression profiles.

There is certain progress in developing gene therapies for GD1. Currently, there is no approved gene therapy for GD2 111. Gene editing is one type of gene therapy that implies making precise alterations to the human genome allowing the one-time treatment for genetic disorders including GD 141. Clustered regularly interspaced short palindromic repeats (CRISPR)/CRISPR associated protein9 (Cas9) (CRISPR/Cas9) system, zinc finger nucleases (ZFNs), as well as transcription activator-like effector nucleases (TALENs) are the most popular and versatile editing platforms 141,142. CRISPR/Cas9 has been specifically employed to create cell models of GD by editing the *GBA1* gene in certain cell lines, providing platforms for pathophysiology studies and drug screening 143. ZFNs have been used in genome editing to increase the levels of lysosomal enzymes. ZFNs are designed to target specific DNA sequences, allowing for precise modifications of the genome. They work by creating double-strand breaks at specific locations, which the cell then repairs, potentially correcting gene mutations 111. Similarly, in case of TALENs, they are engineered to bind to specific DNA sequences, allowing for targeted gene editing. They create breaks in the *GBA1* gene that are then repaired, ideally restoring normal function 111. Although these platforms have been utilized for developing gene editing therapies for GD, none have advanced beyond preliminary studies 111. Noteworthily, CRISPR/Cas9 has been successfully used for developing GD cellular models- human monocytic cell line (THP-1) with *GBA1* mutation as well as glioblastoma cell line (U87) with *GBA1* mutation 143. Additionally, cytosine base editors (CBEs) are enzymes that function as gene editors and can introduce base pair changes (C·G-to-T·A) in genomic DNA. These gene editing enzymes can induce this chemical conversion via enzyme-mediated hydrolytic deamination of cytosine to uracil which is interpreted as thymine by DNA polymerases 144. Generally, these enzymes include the modified CRISPR/Cas9 enzyme which is a naturally occurring cytidine deaminase and an inhibitor of uracil repair. CBEs can be used for gene reversion, thus restoring gene function 144,145.

### 4.5. Allogeneic hematopoietic stem cell transplantation

Allogeneic hematopoietic stem cell transplantation (allo-HSCT) therapy for GD involves transplanting healthy hematopoietic stem cells from a compatible donor into the patient's bone marrow. The transplanted stem cells produce new, healthy blood cells that can break down the accumulated GlcCer in the patient's body. This therapy can potentially halt the progression of the disease and improve the quality of life for GD patients. However, it is a complex and risky procedure that requires careful consideration of the individual situation 146. Besides, the initial achievements of allo-HSCT therapy were quickly overshadowed by the exceptional effectiveness and low toxicity of ERT which remains the most widespread therapy for GD 147.

## 5. Advances in clinics

There are ongoing clinical trials for GD using different treatments. Most of the clinical studies focus on ERT, SRT, and chaperone therapy, or already discussed possible curative treatment methods for GD. Clinical trials have shown promising results for ERT. For example, in the clinical trial (NCT05529992) the side effects of velaglucerase alfa were observed in individuals with GD1 with newly diagnosed disease or currently under treatment with ERT. In another clinical study (NCT04656600) efficacy and safety of treatment with imiglucerase were evaluated in Chinese GD3 patients with neurological manifestations. A clinical trial (NCT03485677) studies the efficacy and safety of eliglustat with or without imiglucerase in pediatric patients with GD1 and GD3. Gene therapy approaches have also offered a potential long-term treatment option for GD. In a clinical study (NCT05324943) gene therapy with FLT201, which is a replication-incompetent single-stranded recombinant AAV vector, is investigated. The study is conducted on GD1 patients for enhancing residual GCase expression and activity thus increasing its potential for the improvement of the clinical phenotype and prevention of cellular GlcCer accumulation. Detailed information on recent clinical trials of GD therapies is given in Supplementary Table 1.

## 6. mRNA/saRNA approach for protein replacement purposes

Along with the global COVID-19 pandemic, a new era of next-generation vaccines, particularly, mRNA vaccines has commenced, offering numerous benefits 20,22,148. mRNA is a molecule that plays a crucial role in protein production. In 1987, Robert Malone successfully transfected the NIH 3T3 mouse cells with the mixture of mRNA and synthetic cationic lipid incorporated into liposome that resulted in the successful expression of the protein 149. Since 1997, Katalin Kariko and Drew Weissman worked together and received the Nobel Prize, recently. They successfully performed the nucleoside modification of mRNA allowing the mRNA to escape innate immune response after delivering into the mice as well as the enhanced translation of the target protein 150. Their approach is successfully utilized in the currently available vaccines of acute respiratory syndrome coronavirus 2 (SARS-CoV-2)- BNT162b2 (Pfizer/BioNTech) and mRNA-1273 (Moderna) 151.

In recent years, researchers have been exploring the use of mRNA as a potential tool for protein replacement therapy. This approach involves the introduction of synthetic mRNA into the patient's cells to produce therapeutic proteins that are missing or defective due to genetic mutations or other factors 25. Once the mRNA enters the cells, it is used as a template to direct the synthesis of a therapeutic protein. The cells then secrete the protein into the bloodstream, where it can travel to the target tissues and exert its therapeutic effects 152,153. mRNA-based protein replacement therapy can be used to treat a wide range of genetic disorders. mRNA-based treatments are also making their way into the field of monoclonal antibody therapies 154. Most importantly, unlike the conventional protein replacement therapies that require the production and purification of large quantities of therapeutic proteins, mRNA-based therapy is much more time-saving. mRNA technology has been studied for protein replacement purposes for certain diseases including heart 155 and alpha 1-antitrypsin deficiency (AATD) 156. There are several clinical trials that use mRNA technology for protein replacement in various diseases including heart failure (NCT03370887), propionic acidemia (NCT04159103), cystic fibrosis (NCT03375047), and ulcers associated with type 2 diabetes mellitus (NCT02935712). This corroborates the idea of mRNA/saRNA technology application for protein replacement therapy for GD. The combination of certain chemical modifications and delivery systems indicates the presence of additional possibilities for optimization 157,158. E.g., N1-methyl-pseudouridine (m1Ψ) nucleoside substitution of uridine in mRNA molecule reduces the detection by the innate immune system, particularly, Toll-like receptors (TLRs) for approximately 100-fold, allowing enhanced protein expression 150,157,159,160.

**Figure 2:**
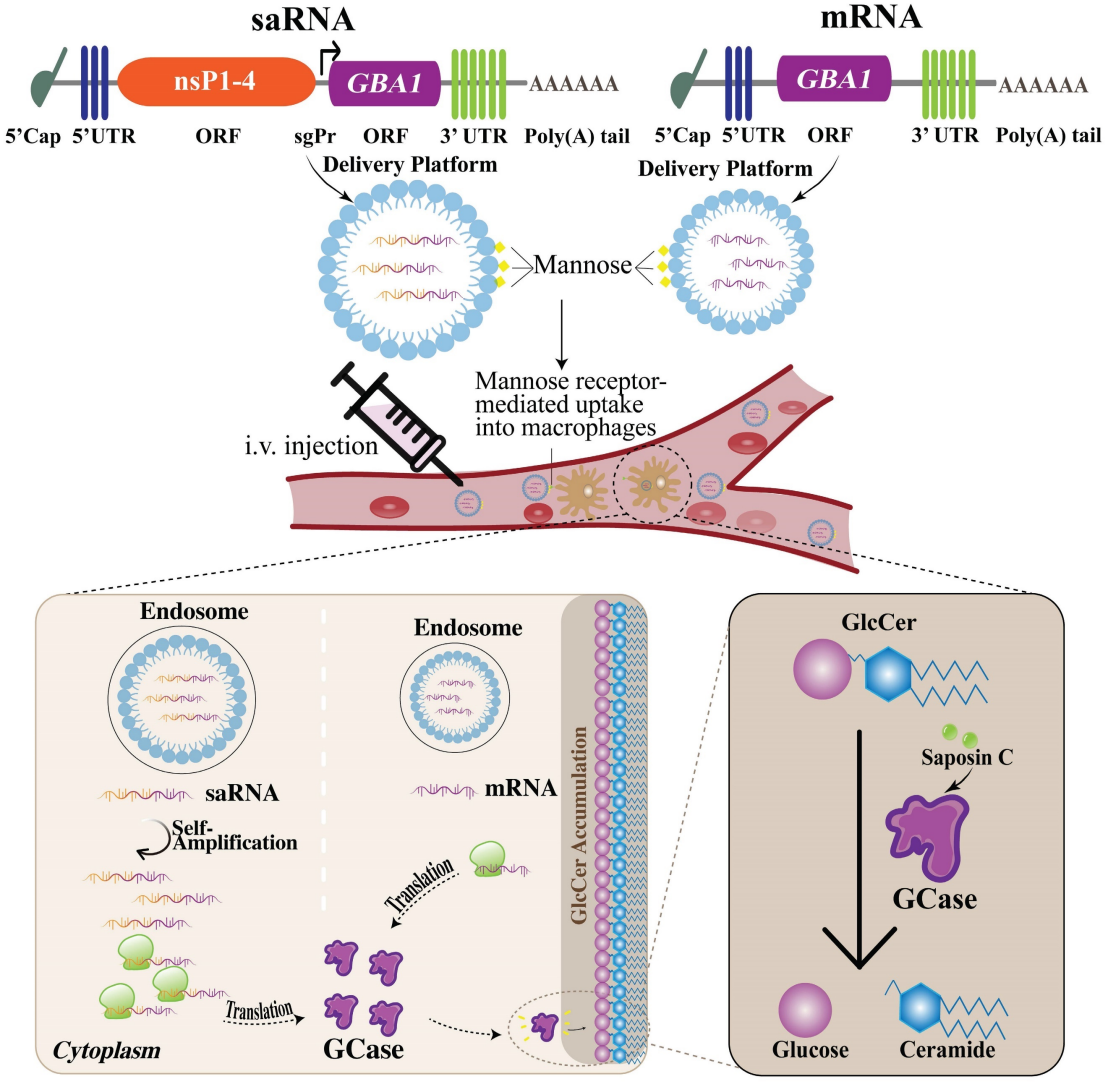
** Application of saRNA and mRNA for GD treatment as protein replacement therapy.** (Left) when the saRNA is injected and delivered into the cells, the translation of replicase takes place. The replicase then uses the saRNA as a template and makes a negative saRNA strand. Then the negative strand saRNA is used as a template by the replicase and self-amplification occurs. Additionally, replicase recognizes sgPr in the negative strand, and sgRNA is synthesized. Consequently, high levels of GCase are produced. (Right) when the mRNA is injected and delivered into the cells, it is translated into GCase protein. Ultimately, in either case, the produced GCase breaks down the GlcCer into glucose and ceramide leading to the normal condition. saRNA, self-amplifying RNA; mRNA, messenger RNA; sgPr, sub-genomic promoter; GD, Gaucher disease; GCase, glucocerebrosidase; GlcCer, glucosylceramide.

The current standard of care for GD is ERT, which involves the administration of recombinant exogenous GCase. However, the limitation of this approach is the need for frequent infusions and the potential for immune reactions to the exogenous protein. The disadvantages of ERT such as invasiveness and inconvenience of infusion along with the short half-time 119,120 might be overcome by the application of mRNA encoding GCase for protein replacement purposes offering better flexibility 161. Another type of RNA molecule- saRNA has recently garnered the attention of researchers as it has the potential to open a new platform in medicine holding significant potential for creating drugs through a simple and cost-effective approach. There are clinical studies ongoing investigating the application of saRNA as a vaccine for severe acute SARS-CoV-2 162,163. saRNA can replicate itself, allowing for the sustained production of therapeutic proteins within the cells. saRNA contains not only the genetic instructions for the production of the protein of interest but also the machinery that is necessary for self-amplification, allowing for the continuous production of therapeutic protein within cells. Particularly, saRNA like mRNA is a positive-strand RNA but unlike non-replicating mRNA, except for the gene of interest (GOI), it contains genes encoding viral replicase. Three types of alphaviruses are usually used in the saRNA approach- Venezuelan Equine Encephalitis, Sindbis, and Semliki Forest Viruses 25. In the saRNA molecule, viral structural proteins are deleted, disabling the production of the infectious virus. In the 5'-3' direction, the saRNA molecule comprises 5'cap, 5'untranslated region (UTR), non-structural proteins (nsPs) 1-4, sub-genomic promoter (sgPr), GOI, 3'UTR, and poly(A) tail. When saRNA is delivered into the cell, nsPs form a replicase, also called an RNA-dependent RNA polymerase (RDRP) which then replicates the whole RNA molecule as well as sub-genomic RNA (sgRNA) resulting in the higher and long-lasting protein expression compared to the conventional mRNA outcome 25. saRNA holds remarkable promise as a potential new tool for protein replacement therapy for GD. Nevertheless, before saRNA application launches in clinics, conventional mRNA should not be forgotten since the more research is done on it, the more is known as well. Therefore, thanks to the rapid development of this strategy, here we propose both- conventional mRNA and relatively new saRNA approaches for GD therapy. The schematic illustration of mRNA/saRNA application for GD treatment is given in Fig. 2.

## 7. Strategy of using mRNA/saRNA approach for GD

**Figure 3:**
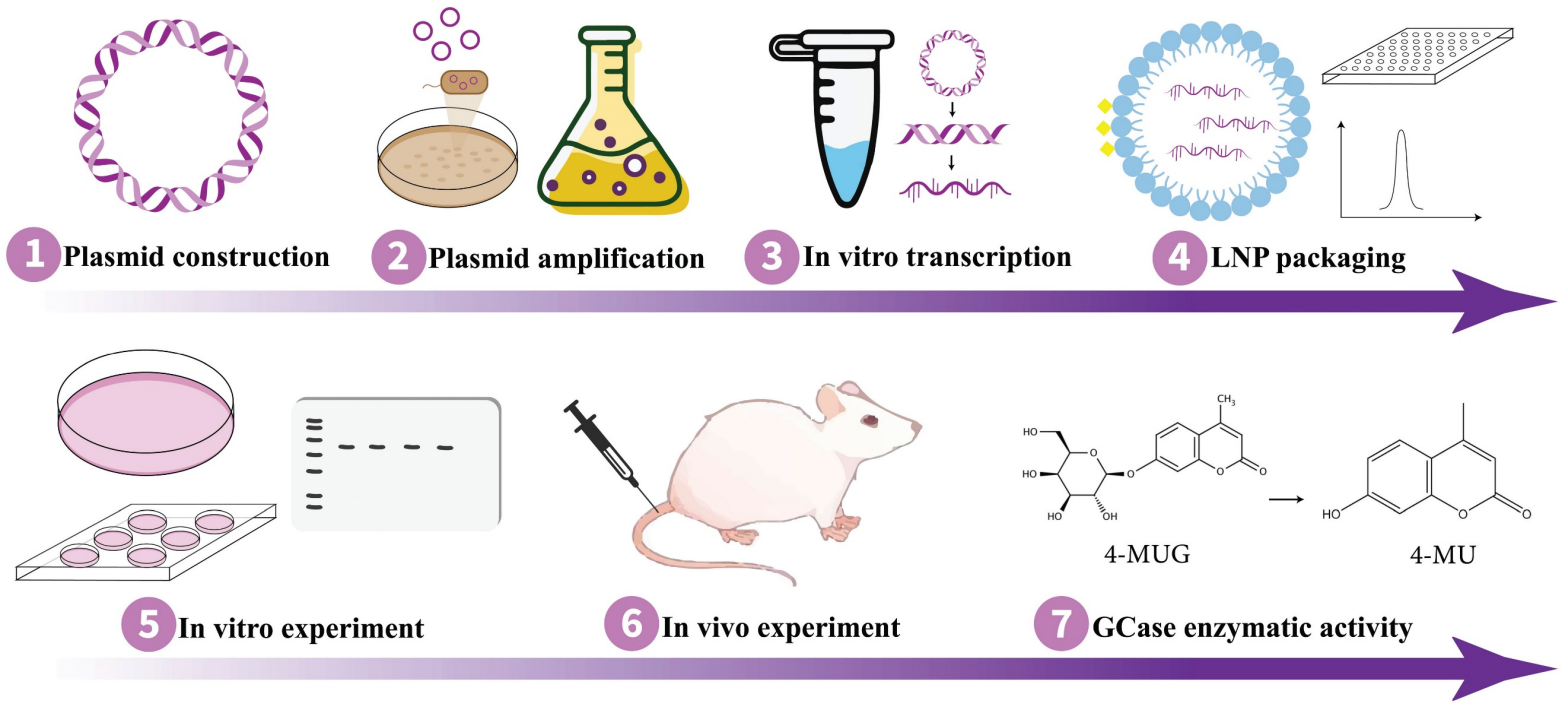
** Schematic of the experimental workflow for studying mRNA/saRNA-based protein replacement therapy in GD.** mRNA, messenger RNA; saRNA, self-amplifying RNA; GD, Gaucher disease.

Implementing the appropriate strategy is essential for achieving the intended results. As mRNA and saRNA represent promising avenues for protein replacement therapies, they hold the potential to expedite and enhance the development of GD treatment for therapy. Remarkably, Moderna's mRNA-3927 has been demonstrated to be generally well-tolerated in phase 1/2 clinical trial with promising outcomes for the other inherited disorder propionic acidemia (NCT04159103) where certain parts of fats and proteins cannot be processed properly 164. In order to proceed pre-clinical study on the application of mRNA/saRNA techniques for protein replacement purposes in GD therapy, it is essential to design an expression plasmid containing the sequence of normal GCase and, in the context of saRNA technology, sgPr. Following the synthesis of the expression plasmid, it needs to be transformed into competent cells, such as DH5α, for amplification. Subsequently, the plasmid DNA should be extracted and purified for further use. To verify GCase expression, the plasmid should be transfected into mammalian cells, such as HEK293T, followed by monitoring for the expression of the target protein. Once the optimal protein expression is confirmed, *in vitro* transcription can be performed to synthesize the mRNA/saRNA. Then the RNA molecule needs to be encapsulated with lipid nanoparticles (LNPs) 165 or another effective delivery system 152,153. Thereafter, validation of GCase protein expression in suitable cells is imperative, which can be followed by the *in vivo* experiment on mouse models. Fig. 3 outlines a workflow for conducting *in vitro* and *in vivo* experiments on GD animal models using mRNA/saRNA technology, delineating the sequential steps from molecular design to therapeutic evaluation.

## 8. Challenges

The primary hurdle in the development of gene therapy for GD has been the absence of an appropriate animal model 166. Fortunately, researchers have successfully developed potential animal models that mimic the neuropathology seen in GD allowing to conduct more studies. Most of the studies use mouse models. For example, Enquist et al., have developed a GD model with Mx1/Cre-loxP system to enable the deletion of GCase exons after birth not to disrupt the GCase activity through the development and skin barrier formation. These mice successfully developed remarkably reduced activity of GCase and significantly elevated GlcCer. Additionally, mice developed infiltration of Gaucher cells in bone marrow, spleen, and liver 167. Mistry et al., have deleted the *GBA1* gene in mouse conditionally using an *Mx1* promoter that resembled the human GD almost entirely 37. Other GD mouse models such as K14-lnl/lnl 168, Tie2-Cre-LoxP 169 also successfully manifested GD symptoms. Besides mouse models, other animal models have been also studied, e.g., Uemura et al., have found that *GBA1^-/-^* medaka (a fish *Oryzias latipes*) can be used as a neuronopathic GD model 170. Cabaso et al., have demonstrated the relevance of using *GBA1b^m/m^* mutant flies for GD studies as they also exhibit remarkably decreased activity of GCase and the accumulation of its substrate 171.

The challenges associated with mRNA-based protein replacement therapy include the necessity of the optimization of mRNA stability, delivery system, translation efficiency, as well as avoidance of innate immune response 172. However, the researchers have made significant progress in the optimization of mRNA sequence and delivery to overcome these challenges 25. LNPs are considered one of the most effective delivery systems which are also employed in FDA-approved mRNA vaccines 173. Furthermore, a small interfering RNA (siRNA) which is the other RNA-based therapeutic (patisiran) approved by FDA also uses LNP as a carrier system 174. Upon entering the host cells, saRNA and its self-replication byproducts induce an innate immune response being recognized as foreign molecules 175. This triggers the release of interferon (IFN) which can interfere with the successful translation of saRNA 176. Besides, the activation of TLRs in the endosomes might be prompted through the endosomal detection mechanisms 20. This will lead to the production of type I IFN along with the other cytokines, resulting in the maturation of dendritic cells (DCs), stimulation of T helper cells and T cell-dependent B cells 176,177. To circumvent the detection of *in vitro* transcribed mRNA and evade the unwanted immune reaction, nucleoside modifications have been applied, a technique refined by Kariko and colleagues. They demonstrated that the inclusion of pseudouridine (Ψ), a modified nucleoside that occurs naturally, into mRNA achieves two outcomes: it diminishes the immune response triggered by the RNA *in vitro* as well as *in vivo*, and it also improves the translation 150. However, this approach is infeasible for saRNA due to its reliance on host cell factors for replication, which would negate the modifications after the initial amplification cycle. Notably, to address this challenge, Blakney et al. have tested saRNA constructs that contained open reading frames (ORFs) of innate inhibiting proteins (IIPs) to see how they affected protein production and immune response. They found that certain proteins, particularly from parainfluenza virus 5 (PIV-5) and Middle East respiratory syndrome coronavirus (MERS-CoV), could significantly increase protein expression and allow the saRNA to evade immune detection 150. Additionally, the fastidious design of mRNA or saRNA constructs necessitates the careful selection of appropriate 5' and 3' UTRs, as well as a signal peptide, along with the addition of the poly-A tail. These elements are crucial for the accurate translation, subsequent secretion of the target protein, as well as stabilization of mRNA construct before translation 178,179.

In order to enhance the effectiveness of LNPs, the incorporation of mannose residues, a process known as mannosylation is also an option. LNPs modified with mannose can improve the uptake ability of macrophages by mannose receptor. Indeed, Goswami et al. claim that mannosylation of LNPs has improved potency for saRNA vaccines 180,181.

A primary obstacle in developing GD therapies utilizing mRNA/saRNA technology lies in the delivery of these molecules to the brain, given the challenges associated with crossing the BBB. BBB is a highly selective and nearly impenetrable protective barrier that separates the blood vessels in the brain from the brain tissue, thereby restricting the entry of various substances including toxins and certain pharmaceuticals, into the brain 182,183. The delivery of drugs across the BBB poses a significant obstacle that hinders the future development of novel therapeutics targeting the brain 184. Nonetheless, some of the large molecules may access the brain via receptor-mediated transport 185. In general, mRNA vaccines, like those developed by Pfizer/BioNTech and Moderna for COVID-19 are not expected to cross the BBB as they are designed to be taken up by cells in the muscle and adjacent lymph nodes at the site of intramuscular (I.M.) injection, with minimal entry into the systemic circulation. However, there is some evidence indicating that small amounts of the mRNA-LNP, particularly, formulated by Moderna for COVID-19 may have the capacity to cross the BBB 186. Gene therapy for GD requires the design of mRNA/saRNA molecules with LNPs to be able to cross BBB and be delivered in the brain, which is challenging. Remarkably, LNPs have been innovatively modified by conjugating monoclonal antibodies (mAbs) to their surface. This modification facilitates the transportation of mRNA into the brain, potentially overcoming the delivery challenges. mAbs ease the vesicular transport of mRNA from one side of a cell to the other side which is called transcytosis. These mAbs are called molecular Trojan horses 187. mAb attaches to a receptor on the BBB, e.g., insulin receptor or transferrin receptor, functioning as a molecular Trojan horse. This facilitates receptor-mediated transcytosis of LNP through the BBB and into the brain extracellular space 187. Notably, multifunctional nanopolymers (MNPs) have been also used for the therapy of CNS owing to the capacity to cross the BBB 188. Remarkably, Sun et al. have studied the application of a CNS-selective delivery system, employing saposin C-dioleoylphosphatidylserine (DOPS) nanovesicles for neuronopathic GD therapy and revealed that the administration of a saposin C-DOPS-GCase successfully delivers functional GCase across a range of tissues, with a pronounced affinity for the brain 189. This method addresses GCase deficiencies within the CNS cells and tissues, demonstrating its effectiveness in ameliorating CNS inflammation and neurological symptoms in a murine model of neuronopathic GD. The research identifies a novel CNS targeting mechanism via a specific phosphatidylserine receptor and the lymphatic system, positioning saposin C-DOPS nanovesicles as a potential new therapeutic avenue for neuronopathic GD 189.

mRNA size usually varies between ~2000-5000 nt while the size of saRNA is much larger- ~15,000 nt 25 and complex that complicates the cellular uptake. This challenge can be surmounted via the application of trans-amplifying RNA (taRNA) which implies the synthesis of saRNA as two separate molecules- mRNA encoding nsPs and mRNA encoding GOI 190,191. Remarkably, taRNA can be as effective as saRNA itself 192. Another obstacle to saRNA approach is the limited clinical data of interactions between nsPs and host factors, the possibility of elevated inflammation due to self-amplification 193, and the generation of innate host immune responses 194,195.

It is also notable that invasive administration of mRNA/saRNA to the brain refers to methods that directly deliver these molecules into the brain tissue or cerebrospinal fluid (CSF), bypassing the BBB. Such methods are considered invasive because they require surgical techniques or other forms of medical intervention that penetrate the protective barriers of the body. Examples of invasive administration routes to the brain include intrathecal injection- delivering substances into the CSF via a lumbar puncture; intracerebroventricular (ICV) injection- injecting directly into the ventricles of the brain; direct parenchymal injection- inserting a needle or catheter directly into the brain tissue, etc. 196. However, these techniques are invasive and painful for patients. Consideration should also be given to potential safety concerns, such as the risk of infection and the possibility of traumatic injuries 197. Besides, the risk of immune response activation in case of both mRNA and saRNA exists that might provoke unintended immune reactions. Moreover, saRNA replication leads to the production of double-stranded RNA intermediates which are known to be highly inflammatory and negatively impact translation efficiency 25. The risk of inducing innate immunity potentially limits repeated administration of saRNA 198. Nonetheless, the benefits of saRNA seem to outweigh the challenges. More research is needed to eliminate these risks to the greatest extent.

The administration routes for mRNA/saRNA therapies include I.V., I.M., as well as intranasal route 199. However, in the context of protein replacement therapy using mRNA or saRNA for GD, the preferred route is I.V. I.V. administration is a common method for systemic delivery, facilitating widespread distribution throughout the body 200. Systemic delivery is important for GD treatment as it targets different organs 199 which are key sites affected by the disease. To circumvent an undesirable immune reaction to administered mRNA, substituting uridines with Ψ is effective 150,159,201. Yet, when multiple injections are required, the development of adaptive immunity and immune memory presents a challenge. When designing mRNA/saRNA constructs, the selection of the appropriate signal peptide is crucial because it significantly influences the final location of the protein once it is translated 179.

## 9. Concluding remarks and future perspectives

In summary, there are two approaches to specifically treat the abnormal accumulation of GlcCer for GD patients, recovering the enzyme activity and reducing the abnormal levels of GlcCer in the lysosome. There are several remedies for enzyme recovery, ERT, gene therapy, allo-HSCT, and chaperone therapy; for reducing the abnormal levels of the substrate, SRT is available.

To date, out of all the mentioned therapies only ERT and SRT are approved by the FDA. Gene editing is supposed to be the future therapy for GD patients. On the other hand, the approval of mRNA vaccines for COVID-19 marks a significant advancement, opening up new opportunities for managing diverse health conditions. This includes its application in cancer prevention and treating genetic diseases. Besides, saRNA technology, with its lower dosage, reduced side effects, and prolonged efficacy thanks to RDRP, is emerging as a superior alternative for protein replacement therapies. Thus, saRNA appears to become a “game changer” in protein deficiency disorders and it fuels great hope for the GD research community. However, the GD research based on the nucleic acid-based technique may be initiated with a conventional mRNA strategy due to its established sophistication and reliability. Then, it may get advanced with saRNA technology leveraging its potential for increased efficacy. Remarkably, GD2 is a rare and severe form of GD and ERT is not sufficiently effective due to the disability to cross the BBB and reach the CNS where the disease primarily affects. This necessitates alternative treatment approaches such as SRT or gene therapy for GD2. Hence, the proposed GCase-mRNA/saRNA technology as a protein replacement therapy for GD patients is intended to be a step forward to aid in therapy development that will be beneficial in terms of prolonging the lifespan and improving the overall quality of life of the GD population. However, it is important to note that certain obstacles, including the right delivery formulations, therapy crossing the BBB, etc., exist in this direction and need to be surpassed. Taken together, mRNA/saRNA with its feasibility, simplicity, and rapid and cost-effective manufacturing technique may lead to the successful development of protein replacement therapeutics for GD.

## Supplementary Material

Supplementary table.

## Figures and Tables

**Table 1:** **GD classification and characteristics.** GD, Gaucher disease; PD, Parkinson's disease; N/A, not applicable.

Clinical features	Non-neuronopathic	Neuronopathic	
		Acute	Chronic
	Type 1	Type 2	Type 3
Incidence	General population- 1 in 40,000-60,000	Less than 1 in 100,000	Less than 1 in 100,000
	Ashkenazi Jews- 1 in 800		
Symptom onset	Any age	Infant	Childhood
Symptoms	Distended abdominal	Dermatological abnormalities	Distended abdominal
	Splenomegaly		Splenomegaly
	Hepatomegaly		Hepatomegaly
	Reduced bone density	Seizures	Seizures
	Bone crises		Oculomotor apraxia
	Reduced lung function	Neck rigidity	Thrombocytopenia
	Reduced platelet count		Cognitive problems
	Thrombocytopenia	Oculomotor paralysis	Blood disorders
	Anemia/cytopenia		Respiratory disorders
	Nosebleeds/bruising	Swallowing disorders	Anemia
	Abdominal discomfort		Bone disease
Life expectancy	Childhood/adulthood	Before 2 years	Childhood/early adulthood
Disease course	Progressive	Rapidly progressive	Progressive
Associations with other diseases	Osteonecrosis	N/A	Osteonecrosis
	Osteoporosis		Osteoporosis
	PD		PD
	Neoplasia		Neoplasia
	Cirrhosis		Cirrhosis
Treatment	ERT/SRT	None/hematopoietic stem cell transplantation	ERT/none
Common *GBA1* mutations	N370S	Diverse	L444P

**Table 2:** ** FDA-approved drugs for treating GD.** FDA, Food and Drug Administration; GD, Gaucher disease; ERT, enzyme replacement therapy; SRT, substrate reduction therapy; I.V., intravenous.

Therapy class	ERT			SRT	
Brand name	Cerezyme	Elelyso	VPRIV	Zavesca	Cerdelga
Manufacturer	Genzyme	Protalix, Pfizer	Takeda (Shire plc)	Actelion Pharmaceuticals	Sanofi Genzyme
Drug name	Imiglucerase	Taliglucerase alfa	Velaglucerase alfa	Miglustat	Eliglustat
Molecular formula	C_2532_H_3854_N_672_O_711_S_16_	C_2580_H_3918_N_680_O_727_S_17_	C_2532_H_3850_N_672_O_711_S_16_	C_10_H_21_NO_4_	C_23_H_36_N_2_O_4_
Manufacture	CHO cells	Carrot cells	Human fibrosarcoma cells	Chemical synthesis	
Dosing (average)	Should be individualized: 2.5-60 U/kg	60 U/kg	60 U/kg	100 mg	84 mg
Administration interval (average)	Once every other week	Once every other week	Once every other week	Once a day	Once/twice a day
Administration route	I.V. injection	I.V. injection	I.V. injection	Oral	Oral
Mechanism of action	Supplies the mutated GCase activity by increasing the concentration of exogenous GCase			Reduces GlcCer by inhibiting GlcCer synthase	
Main side effects	Abdominal discomfort, nausea, vomiting, diarrhea	Chest tightness, dizziness, feeling of warmth, hives or welts, itching, skin rash	Headache, arthralgia, cough, difficulty with breathing, fever, dizziness, abdominal pain	Diarrhea, stomach pain/bloating, weight loss, upset stomach, vomiting	Fatigue, headache, nausea, diarrhea, back pain, and muscle ache
Average annual cost (USD)	384,814 to 705,493	185,940 to 354,977	152,909 to 560,666	~98,000	253,675 to 507,350
FDA approval date	May 23, 1994	May 1, 2012	February 26, 2010	July 31, 2003	August 19, 2014
