# Supplementary material for: A review on Gaucher disease: therapeutic potential of β-glucocerebrosidase-targeted mRNA/saRNA approach
Source: Int J Biol Sci. 2024 Mar 17;20(6):2111–29. doi: 10.7150/ijbs.87741 (PMC11008270; doi:10.7150/ijbs.87741)
Supplement: Supplementary file 1 — Supplementary table. [file ijbsv20p2111s1.pdf]

Supplementary Table 1. Recent clinical trials of GD (since January 1, 2018). Source: ClinicalTrials.gov. Search criteria: Gaucher Disease; interventional studies; phase 2,3,4, N/A; (Accessed in May 2023, sorted from the newest to the oldest start dates). GD, Gaucher disease; ERT, enzyme replacement therapy; SRT, substrate reduction therapy; PD, Parkinson's disease; N/A, not applicable.

Drug type	Drug name / treatment	Condition	Country	Sponsors and collaborators	(Estimated) enrollment	Status	Start date	(Estimated) end date	Phase	NCT number
ERT	Velaglucerase Alfa	GD	China	Takeda	20	Recruiting	January 3, 2023	December 4, 2024	3	NCT05529992
	CAN103	GD1 GD3	China	CANbridge (Suzhou) Bio-pharma Co., Ltd.	40	Recruiting	July 11, 2022	December 30, 2024	1/2	NCT05447494
	Cerezyme®/ Imiglucerase	GD	China	Sanofi	10	Active, not recruiting	March 2, 2021	October 18, 2023	4	NCT04656600
	Taliglucerase alpha	GD3	India Israel Turkey	Ari Zimran Pfizer	14	Active, not recruiting	November 20, 2020	August 1, 2023	4	NCT04002830
	Elelyso 60 units/kg	GD1	US	Pfizer	0	Withdrawn	July 31, 2019	June 30, 2020	4	NCT03021941
	Velaglucerase alfa	GD1	Israel	Shaare Zedek Medical Center	15	Completed	September 4, 2018	March 18, 2021	4	NCT03702361
Pharmacological Chaperone Therapy	Ambroxol	GD1	Israel	Shaare Zedek Medical Center	40	Completed	March 1, 2019	December 30, 2022	2	NCT03950050
	Arimoclomol	GD1, GD3	India	ZevraDenmark	39	Terminated	June 6, 2018	October 15, 2021	2	NCT03746587
SRT	Cerdelga and Cerezyme	GD3	Taiwan	National Taiwan University Hospital Sanofi	4	Completed	April 23, 2018	September 11, 2020	N/A	NCT03519646
	Eliglustat GZ385660 Imiglucerase GZ437843	GD1 GD3	Argentina Canada France Italy Japan Russian Federation Spain Sweden	Sanofi	57	Active, not recruiting	April 11, 2018	November 20, 2025	3	NCT03485677

			Turkey<br>UK							
	Venglustat<br>Imiglucerase	GD3	US<br>Argentina<br>China<br>Canada<br>France<br>Germany<br>Hungary<br>Japan	Genzyme, a Sanofi Company	40	Recruiting	April 18, 2022	January 29, 2026	3	NCT05222906
	Venglustat	GD3	US<br>Germany<br>Japan<br>United Kingdom	Genzyme, a Sanofi Company	13	Active, not recruiting	January 4, 2017	September 30, 2025	2	NCT02843035
Gene Therapy	FLT201	GD1	Argentina<br>Brazil<br>Germany<br>Israel<br>Italy<br>Paraguay<br>Spain<br>United Kingdom	Freeline Therapeutics	18	Recruiting	April 15, 2022	January 31, 2025	1/2	NCT05324943
	AVR-RD-02	GD1	US	AVROBIO	16	Recruiting	May 30, 2019	December 2023	1/2	NCT04145037
	LY3884961<br>Methylprednisolone<br>Sirolimus<br>Prednisone	GD2	US<br>UK	Prevail Therapeutics<br>Eli Lilly and Company	15	Recruiting	June 29, 2021	September 2028	1/2	NCT04411654
	LY3884961<br>Methylprednisolone<br>Sirolimus	PD	US<br>Israel	Prevail Therapeutics<br>Eli Lilly and Company	20	Recruiting	January 3, 2020	April 2028	1/2	NCT04127578
	LY3884961<br>Methylprednisolone	Peripheral manifestat	US	Prevail Therapeutics<br>Eli Lilly and Company	15	Recruiting	December 20, 2022	September 11, 2030	1/2	NCT05487599

	Sirolimus<br>Prednisone	ions of<br>GD								
Antibody Drugs	Ledipasvir/<br>Sofosbuvir	GD<br>HCV	Egypt	Mansoura University Children Hospital	10	Unknown	April 3, 2018	October 2019	4	NCT03721627
Digital Application	Digital Engagement Application (GD App)	GD1	US	Takeda	4	Completed	April 22, 2021	February 16, 2023	4	NCT04718779
	Zamplo Digital Health Platform	Metabolic Disease<br>Fabry Disease<br>GD<br>Pompe Disease<br>Mitochondrial Diseases	Canada	M.A.G.I.C. Clinic LTD<br>Hanalytics Solutions Inc.	150	Unknown	August 7, 2020	November 30, 2021	N/A	NCT04758130
